# Spectroscopic and crystallographic characterization of two cathinone derivatives: 1-(4-fluorophenyl)-2-(methylamino)pentan-1-one (4-FPD) hydrochloride and 1-(4-methylphenyl)-2-(ethylamino)pentan-1-one (4-MEAP) hydrochloride

**DOI:** 10.1007/s11419-017-0393-6

**Published:** 2017-11-17

**Authors:** Marcin Rojkiewicz, Piotr Kuś, Joachim Kusz, Maria Książek

**Affiliations:** 1 Department of Organic Synthesis, Institute of Chemistry, University of Silesia, 9 Szkolna Street, 40-006 Katowice, Poland; 2 Department of Crystal Physics, Institute of Physics, University of Silesia, 4 Uniwersytecka Street, 40-007 Katowice, Poland

**Keywords:** 4-FPD, 4-MEAP, Mass spectrometry, X-ray crystallography, Infrared and Raman spectroscopies, NMR spectroscopy

## Abstract

**Purpose:**

In this study, we performed identification and physicochemical characterization of two cathinone derivatives, 4-FPD and 4-MEAP, found in market-available materials.

**Methods:**

The compounds were characterized by electrospray ionization ion trap mass spectrometry (MS) in MS^2^ and MS^3^ modes, gas chromatography–MS, infrared, Raman and ultraviolet-visible spectroscopies, X-ray crystallography, differential scanning calorimetry and nuclear magnetic resonance spectroscopy.

**Results:**

We could obtain detailed and comprehensive physicochemical characterization of 4-FPD and 4-MEAP—new cathinone derivatives available on the designer drugs market.

**Conclusions:**

Dynamic growth in the number of psychoactive substances available on the designer drug markets makes it compulsory to obtain analytical data allowing unequivocal identification of these drugs in the fastest possible way. In this study we presented analytical data useful in quick identification of the investigated compounds.

**Electronic supplementary material:**

The online version of this article (10.1007/s11419-017-0393-6) contains supplementary material, which is available to authorized users.

## Introduction

New psychoactive substances (NPS) have been progressively replacing classical narcotics on the illegal drug market. Among most prominent compounds of this type are synthetic cathinones and synthetic cannabinoids. Because of widespread abuse of such designer drugs (“legal highs”), the associated health risks and high frequency of novel drug variants constantly emerging on the market, numerous studies of these psychoactive substances were undertaken in recent years [1–7].

Synthetic cathinones are a broad group of “designer drugs“. Biological effects of their action are similar to those caused by amphetamine or cocaine [8, 9]. They are marketed either as collectibles (“not suitable for human consumption”) or as “research chemicals”. Synthetic cathinones are akin to amphetamines, and the only difference between synthetic cathinones and the respective amphetamines is the presence of a carbonyl group in the β-position with respect to the amino group in cathinone derivatives. For this reason, synthetic cathinones are called bk-amphetamines. Since cathinone is a β-keto derivative of amphetamine, it stimulates and shows sympathomimetic action upon the central nervous system. The action of cathinone and its derivatives relies mainly on inhibiting reuptake of neurotransmitters; this results in their increased concentration in the inter-synaptic space and stronger activation of cathinone-sensitive receptors. Additionally, relatively strong inhibitory actions of cathinone derivatives towards monoamine oxidase (MAO) and higher selectivity of the cathinone derivatives toward MAO-B isoenzyme have been reported [10]. Inhibition of the isoenzyme, normally metabolizing dopamine, prevents degradation of the important neurotransmitter. It can be assumed that synthetic cathinones show affinity towards monoamine transporters responsible for endogenous amines, such as serotonin or dopamine, by increasing their concentration in inter-synaptic space [11, 12]. Health effects of various market-available synthetic cathinones can differ. Peripheral nervous system effects of cathinone derivative abusers include dilated pupils, elevated blood pressure or hyperthermia. Psychoactive effects of these compounds include states of increased excitement, elevated assertiveness, euphoria, better concentration, anxiety, restlessness or hallucinations. Withdrawal symptoms include sleeplessness, irritability, concentration problems, depression and various psychoses. Long-term abuse of cathinone derivatives may be destructive for both nervous as well as blood vascular systems and may result in irreversible mental disorders. The literature reports and subjective accounts of cathinone derivative abusers have cited occurrence of panic attacks, convulsions, hallucinations, aggressive behavior, chest pain, hypothermia and many other symptoms, which are sometimes life-threatening [1, 13–18].

**Fig. 1:**
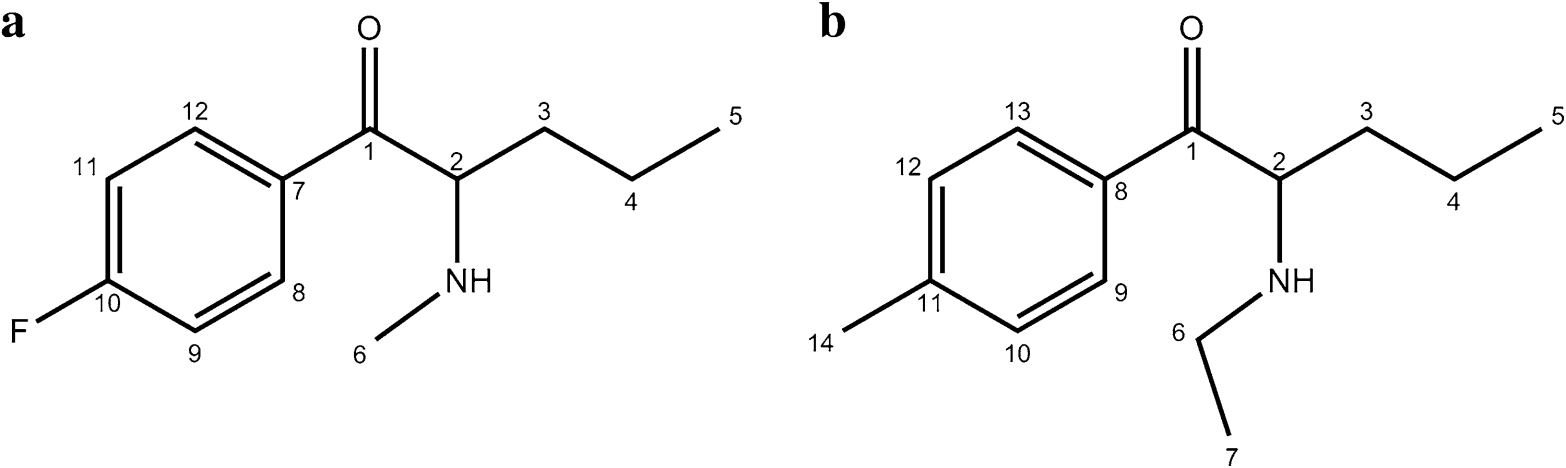
Structures of 1-(4-fluorophenyl)-2-(methylamino)pentan-1-one (4-FPD) (**a**) and 1-(4-methylphenyl)-2-(ethylamino)pentan-1-one (4-MEAP) (**b**)

In this study, we performed identification and physicochemical characterization of two cathinone derivatives, 4-FPD and 4-MEAP (Fig. 1), found as market-available materials. To characterize both substances, data were obtained by electrospray ionization/ion trap mass spectrometry (MS) in MS^2^ and MS^3^ modes, gas chromatography–mass spectrometry (GC–MS), infrared (IR), Raman and ultraviolet-visible (UV-VIS) spectroscopies, X-ray crystallography, differential scanning calorimetry (DSC) and nuclear magnetic resonance (NMR) spectroscopy. To our knowledge, this is the first comprehensive report detailing identification and characterization of 4-FPD and 4-MEAP, two cathinones available on the NPS market, though some selected data for 4-MEAP have been available previously [19, 20].

## Materials and methods

### Chemicals

All reagents used were of the HPLC and MS grade. Water (Chromasolv), methanol, deuterated dimethyl sulfoxide (DMSO-*d*_6_ for NMR analysis) were purchased from Sigma-Aldrich (Poznań, Poland).

### Sample preparation

The analyzed samples were purchased in Poland in mid-2017 from an Internet vendor. Both samples were delivered in powdered forms, which were found pure without any additive by some spectroscopies and chromatographies. The samples were sold as “4-FPD” (compound **1**) and “4-methyl pentedrone” (compound **2**) and were thus analyzed without any purification. For gas chromatography studies, a 10-mg aliquot of each sample was dissolved in 1 mL methanol by ultrasonication (10 min). Each 10 µL aliquot of such solution was collected and diluted 100-fold with methanol and analyzed. For NMR spectroscopic analysis, 10 mg of the powder sample was dissolved in 0.6 mL DMSO-*d*_6_. For DSC, IR, Raman and UV-VIS studies, 5 mg of each sample was taken. The DSC, IR and Raman analyses were performed without any further sample treatment, and for the UV-VIS analysis the samples were dissolved in methanol, diluted tenfold with methanol before analysis.

### GC–MS analysis

For GC**–**MS analysis, the Thermo Trace Ultra chromatograph was used, coupled with the Thermo DSQ mass spectrometer (Thermo Scientific, Warsaw, Poland). The analyses were carried out using the Rxi^®^-5Sil MS column (30 m × 0.25 mm i.d., 0.25 μm film thickness; Restek, Bellefonte, PA, USA). The following working parameters were employed: injector temperature, 260 °C; oven temperatures, 100 °C for 2 min, ramp at 20 °C/min to 260 °C; carrier gas (helium) flow rate, 1.2 mL min^−1^; MS transfer line temperature, 250 °C; MS source temperature, 250 °C; injection volume, 1 μL in the splitless mode. For structural confirmation, electron ionization (EI) mass spectra were matched to an EI-MS library [21].

### Electrospray ionization–mass spectrometry^*n*^

A Varian MS-500 mass spectrometer with electrospray ionization source (Varian Inc., Palo Alto, CA, USA) was used. The obtained data were processed using MS Workstation software (Varian Inc.). The analytes were electrosprayed in the positive mode (ESI(+)-MS). Fragmentation in the ESI-MS^2^ and ESI-MS^3^ mode was carried out in the scanning range of *m*/*z* 50–500. The source temperature was 350 °C, and the carrier and ionizing gases were nitrogen and helium, respectively.

### NMR spectroscopy

The NMR spectra were recorded using UltraShield 400 MHz apparatus (Bruker, Bremen, Germany) with DMSO-*d*_6_ as solvent. The data were collected with the chemical shifts referenced to a residual solvent signal.

### Fourier transform infrared, Raman and UV-VIS spectroscopies

Each IR spectrum (wavenumber range 3500–400 cm^−1^) of each evidence material was acquired using a Nicolet iS50 FT-IR spectrometer (Thermo Scientific) and the attenuated total reflectance technique. Raman measurements were performed using a Thermo Scientific™ DXR™2xi Raman imaging microscope equipped with a 780 nm laser (Thermo Scientific). UV-VIS absorption spectra of methanol-dissolved samples were recorded using the PerkinElmer Lambda Bio 40 UV-Vis spectrometer (*λ* = 200–1000 nm) (PerkinElmer, Kraków, Poland).

### Differential scanning calorimetry

DSC was performed with a TA-DSC 2010 apparatus (TA Instruments, New Castle, DE, USA) under nitrogen using aluminum sample pans in a temperature range from 20 °C to over the clearing point.

### X-ray spectroscopy

The single crystal X-ray experiments for both compounds were performed at 100 K. The data were collected using a SuperNova kappa diffractometer with Atlas CCD detector (Agilent Technologies, Santa Clara, CA, USA). For the integration of the collected data, the CrysAlis^Pro^ software (version 1.171.38.41q, 2015; Rigaku Oxford Diffraction, Sevenoaks, UK) was used. The solving and refining procedures were similar for both compounds. The structures were solved using direct methods with the SHELXS97 software, and the solutions were refined using SHELXL-2014/7 program [22]. CCDC 1573894-1573895 contains supplementary crystallographic data for this paper. These data can be obtained free of charge from The Cambridge Crystallographic Data Centre via: http://www.ccdc.cam.ac.uk/data_request/cif.

## Results and discussion

### GC–MS and ESI-MS^*n*^

**Fig. 2:**
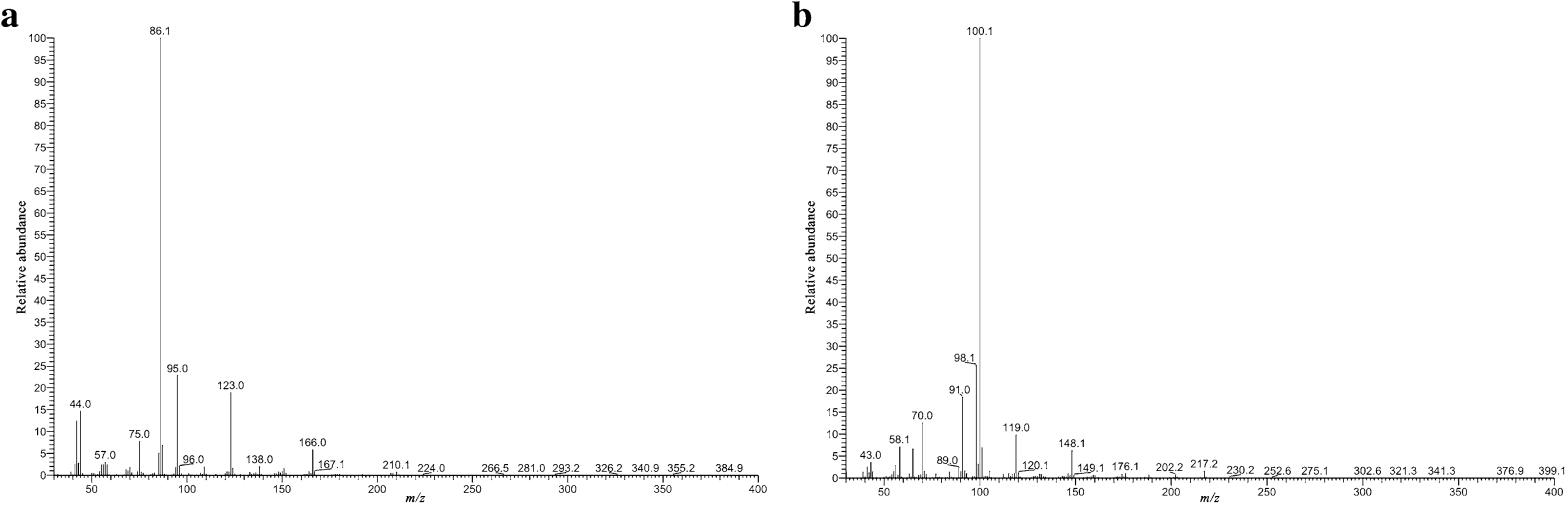
Gas chromatography–electron ionization-mass spectrometry (GC–EI-MS) spectra of compounds **1** (**a**) and **2** (**b**)

The samples were analyzed by GC–MS, and the resulting mass spectra of compounds **1** and **2** are shown in Fig. 2. The spectra were subjected to examination whether they hit specific compounds in the EI-MS library; as results compounds **1** and **2** were found to be possibly 4-FPD and 4-MEAP, respectively.

**Fig. 3:**
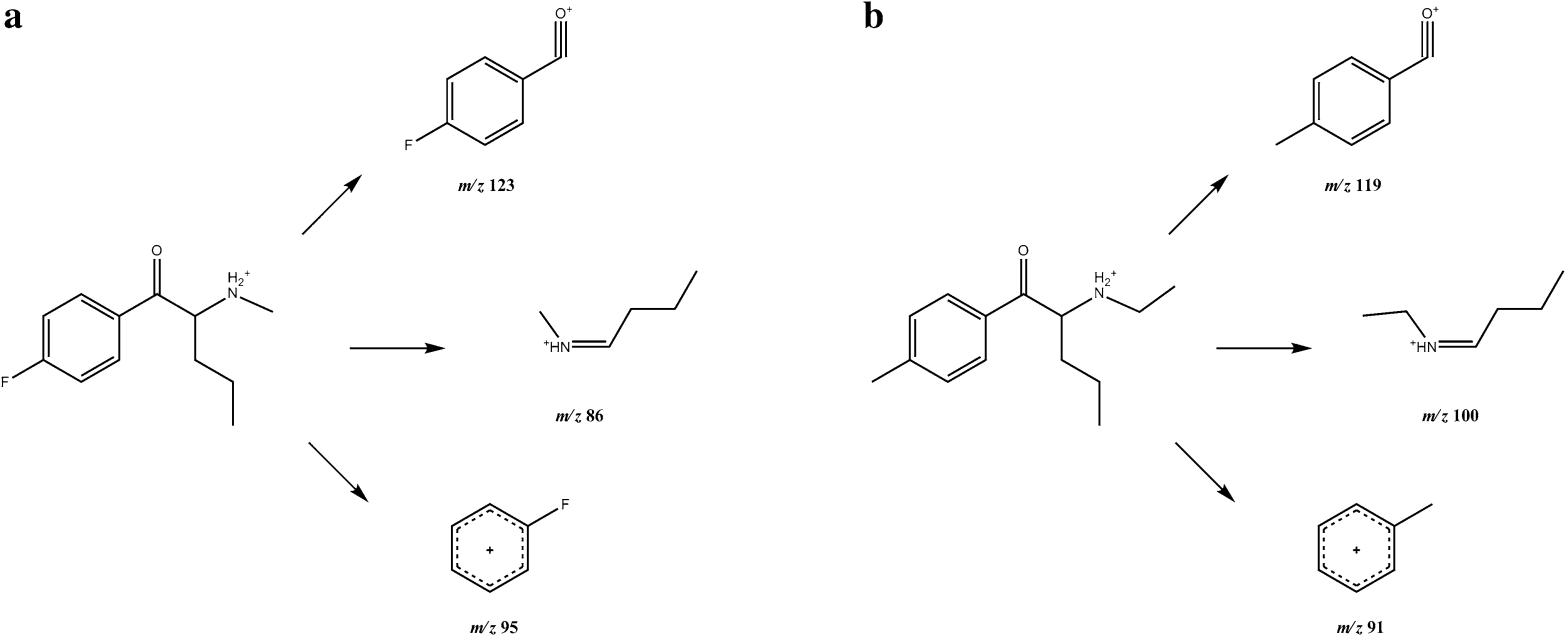
GC–EI-MS fragmentation pathways of compounds **1** (**a**) and **2** (**b**)

In the mass spectra obtained in the EI-MS mode, each main fragment ion was detected for compounds **1** and **2** at *m*/*z* 86 and 100, respectively. Other less intense fragments present in the spectra were at *m*/*z* 95 and 123 for 4-FPD (**1**) and at *m*/*z* 91 and 119 for 4-MEAP (**2**). Possible structures of the fragmentation products derived from the parent structures of compounds **1** and **2** are presented in Fig. 3, and were in accordance with reported fragmentation pathways of other cathinone derivatives [23]. The obtained spectra were almost identical with those originating from 4-FPD and 4-MEAP standards [20, 21]. Also, comparison of our data with those for a well-known stimulant pentedrone has revealed the presence of the same base peak (*m/z* 86) in the case of compound **1**, which suggests bond cleavage between carbon atoms 1 and 2 (carbon numbering shown in Fig. 1). The difference of 14 Da between *m*/*z* 86 (4-FPD and pentedrone) and *m*/*z* 100 (4-MEAP) corresponds well with the presence of an additional methyl group in compound **2**. Furthermore, other differences in *m/z* values seen in the mass spectra of compounds **1** and **2** result from the presence of different moieties, as compared to pentedrone, confirming validity of the proposed fragmentation pathways.

**Fig. 4:**
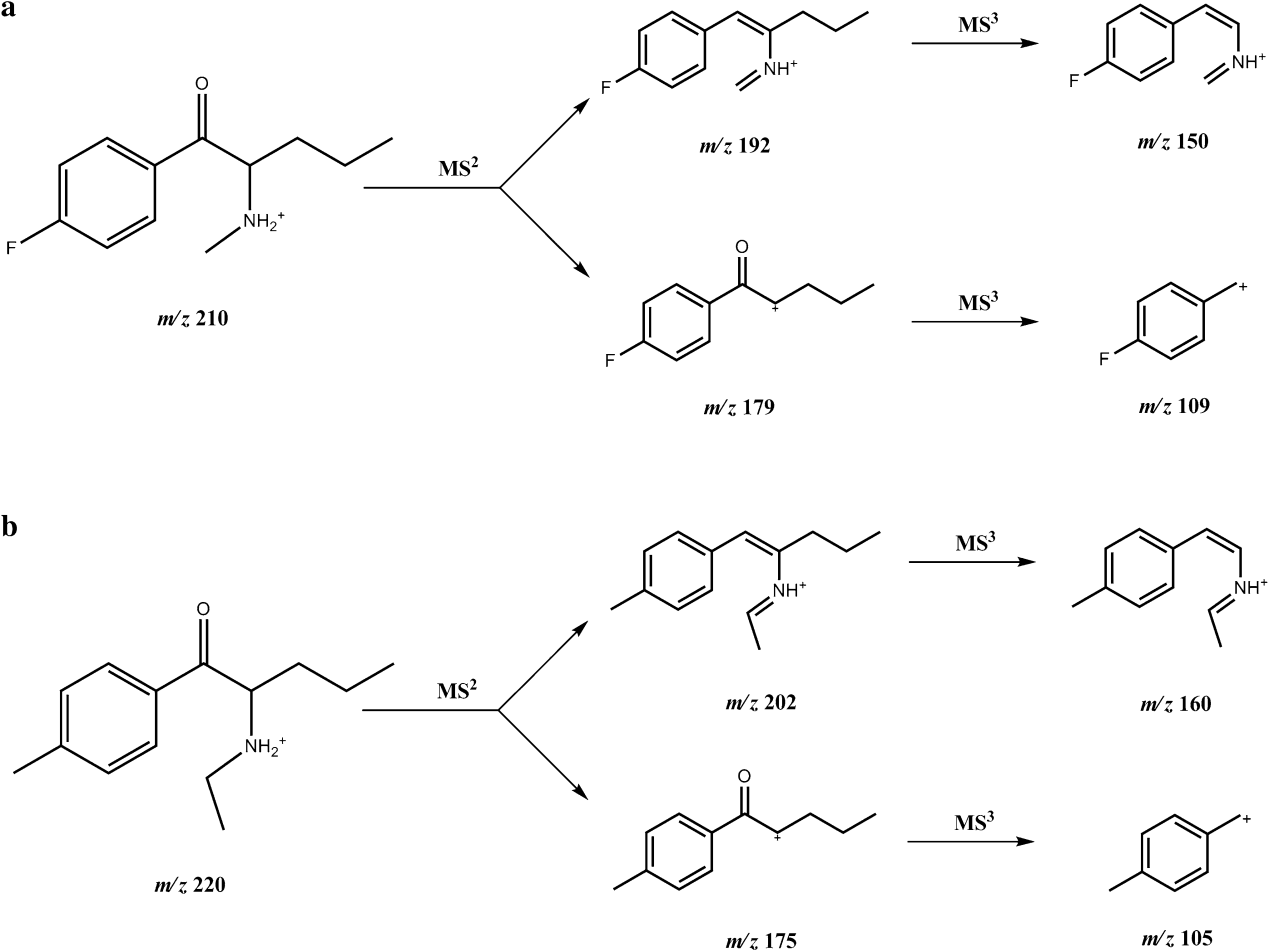
Electrospray ionization-MS (ESI-MS) fragmentation pathways of compounds **1** (**a**) and **2** (**b**) in MS^2^ and MS^3^ modes

In the ESI-MS spectrum, the protonated molecule [M+H]^+^ was seen at *m/z* 210 for compound **1** (4-FPD) and at *m/z* 220 for compound **2** (4-MEAP). The samples were also analyzed by tandem and triple stage mass spectrometry (MS^2^ and MS^3^), using direct infusion. In the MS^2^ mode, elimination of a water molecule for both compounds was observed [M+H-H_2_O]^+^, which is characteristic for cathinone derivatives [24]. The probable fragmentation pathways in the MS^2^ and MS^3^ modes for **1** and **2** are presented in Fig. 4. The above analyses of the spectra by both EI-MS and ESI-MS^*n*^ further support the idea that compounds **1** and **2** are 4-FPD and 4-MEAP, respectively.

### ^1^H and ^13^C NMR spectroscopy

NMR spectroscopy was employed to confirm the structures of investigated compounds. The data for compounds **1** and **2** are presented in Tables 1 and 2, respectively (atom numbering according to Fig. 1).

**Table 1:** ^1^H and ^13^C nuclear magnetic resonance (NMR) data for compound **1** (4-FPD)

Atom position	Carbon chemical shifts (ppm)	Proton chemical shifts (ppm)
1	195.31	–
2	62.31	5.26 (t, *J* = 5.3 Hz, 1H)
3	31.98	1.78–1.87 (m, 1H); 1.90–1.98 (m, 1H)
4	31.78	1.02–1.15 (m, 1H); 1.26–1.38 (m, 1H)
5	14.10	0.78 (t, *J* = 7.3 Hz, 3H)
6	17.54	2.56 (s, 3H)
7	131.30	–
8, 12	132.37; 132.47 (*J*_C–C–C–F_ = 9.8 Hz)	8.17 (m, 2H)
9, 11	116.51; 116.53 (*J*_C–C–F_ = 22.1 Hz)	7.45 (m, 2H)
10	165.02; 167.55 (*J*_C–F_ = 254.4 Hz)	–
N–H	–	9.59 (bs, 2H)

The numbering of carbon atoms is given in Fig. 1

**Table 2:** ^1^H and ^13^C NMR data for compound **2** (4-MEAP)

Atom position	Carbon chemical shifts (ppm)	Proton chemical shifts (ppm)
1	196.26	–
2	60.72	5.23 (t, *J* = 5.2 Hz, 1H)
3	32.36	1.82–1.98 (m, 2H)
4	17.70	1.01–1.34 (m, 1H); 1.22–1.37 (m, 1H)
5	11.56	0.77 (t, *J* = 7.3 Hz, 3H)
6	41.59	2.84–2.92 (m, 1H); 2.97–3.06 (m, 1H)
7	14.14	1.27 (t, *J* = 7.2 Hz, 3H)
8	132.17	–
9, 13	129.33	7.99 (d, *J* = 8.2 Hz, 2H)
10, 12	130.18	7.41 (d, *J* = 8.0 Hz, 2H)
11	145.93	–
14	21.73	2.87 (s, 3H)
N–H	–	9.14 (bs); 9.74 (bs)

The numbering of carbon atoms is given in Fig. 1

Because of interaction of protons with fluorine in the 4-FPD molecule, instead of a classical set of signals in ^1^H NMR spectrum in the aromatic region (i.e., dd), a number of multiplets were observed for each group of equivalent protons. Also in the ^13^C NMR spectrum, the coupling of carbon with the fluorine atom could be observed that explains splitting in the carbon signals (Table 1). The ^1^H and ^13^C NMR spectra for compounds **1** and **2** are available in the electronic supplementary material (Figs. S1–S4). The NMR data confirmed the identification of compounds **1** and **2** to be 4-FPD and 4-MEAP, respectively.

### IR, Raman and UV-VIS spectra

IR and Raman techniques can be useful in characterizing designer cathinones, especially identifying characteristic functional groups. In the IR spectra, a strong carbonyl stretch at 1688 cm^−1^ was observed for both compounds. Also, the aliphatic and aromatic C–H stretching at 2700–3000 cm^−1^ and the amine hydrochloride salt bands can be observed for both compounds. Characteristic C–C vibrations in an aromatic ring appeared at 1598 and 1605 cm^−1^ for 4-FPD (**1**) and 4-MEAP (**2**), respectively. In the Raman spectroscopy, strong C=O bands were observed at 1690 cm^−1^ for both compounds. The C–C aromatic bands in the Raman spectra gave actually the same wavenumber values as in the IR spectra. Both IR and Raman spectra were very similar for compounds **1** and **2**. This is probably due to structural similarity of the investigated compounds. The IR and Raman spectra for compounds **1** and **2** are available in the electronic supplementary material (Figs. S5–S8). The UV spectra recorded by UV-VIS spectrometry showed absorption maxima at 253 and 265 nm for the compounds **1** and **2**, respectively.

### Melting points

Melting points for compounds **1** (4-FPD hydrochloride) and **2** (4-MEAP hydrochloride) obtained by DSC analysis were 245 and 223 °C, respectively; those measured in the classical way were 243–245 °C and 220–222 °C, respectively. Both compounds melted with decomposition.

### X-ray studies

**Fig. 5:**
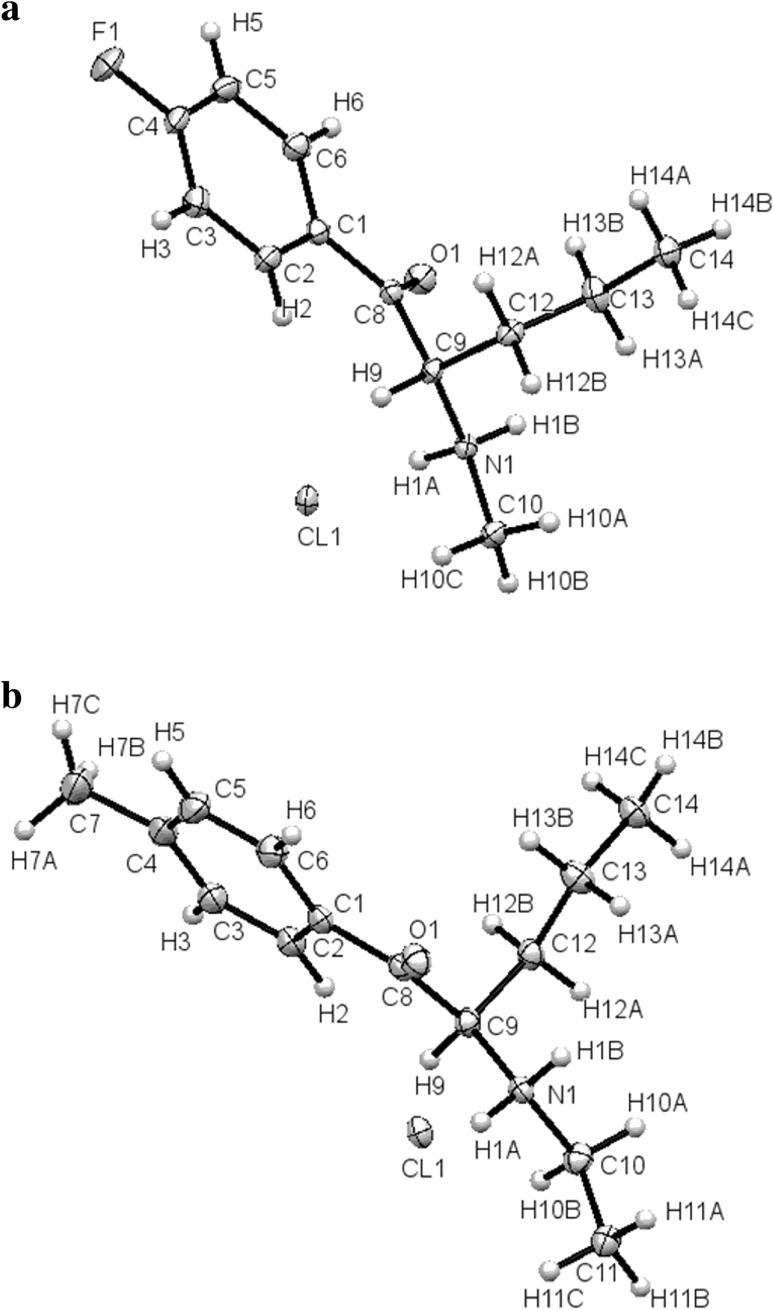
Molecular structures of compounds **1** (4-FPD hydrochloride) (**a**) and **2** (4-MEAP hydrochloride) (**b**), showing atom-labeling schemes. Ellipsoids representing displacement parameters are drawn at the 50% probability level

**Fig. 6:**
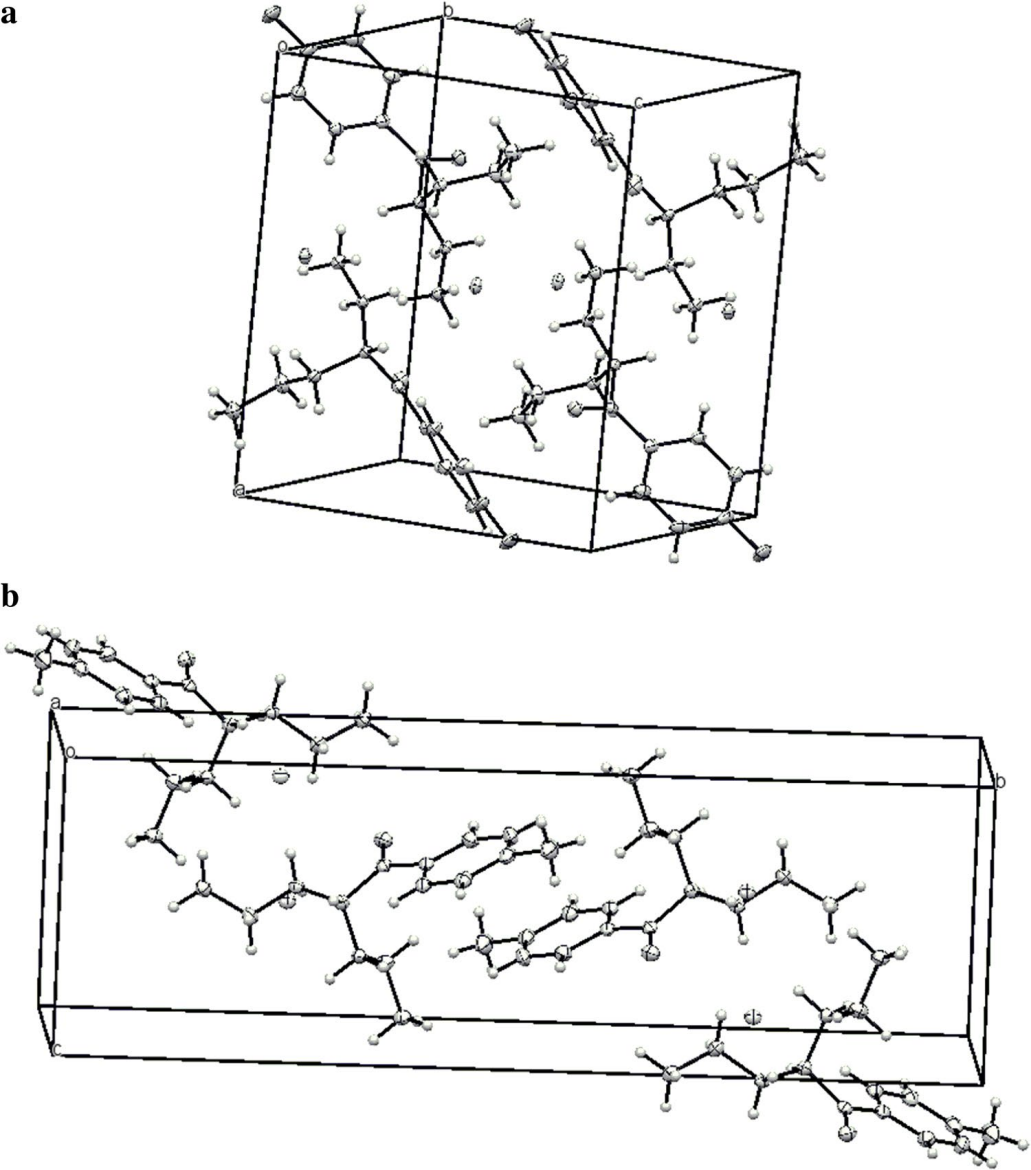
Packing diagram of compounds **1** (4-FPD hydrochloride) (**a**) and **2** (4-MEAP hydrochloride) (**b**)

Crystal data and structure refinements for both compounds are summarized in Table 3. The molecular structures of compounds **1** (4-FPD hydrochloride) and **2** (4-MEAP hydrochloride) are shown in Fig. 5. Packing diagrams for both compounds are shown in Fig. 6. All distances, angles and planarity of ring systems are typical. In the crystals of both compounds, a racemic mixture was present. Compounds **1** and **2** have two enantiomeric ion pairs in the unit of the crystal lattices.

**Table 3:** Crystal data and structure refinements for compounds **1** (4-FPD) and **2** (4-MEAP)

	Compound **1**	Compound **2**
Molecular formula	C_12_H_17_FNOCl	C_14_H_22_NOCl
Molecular weight	245.71	255.78
Crystal system	Monoclinic	Monoclinic
Space group	*P2*_*1*_ */c*	*P2*_*1*_ */n*
Temperature (*K*)	100	100
*a* (Å)	11.9884 (2)	7.1344 (2)
*b* (Å)	9.6621 (2)	24.7843 (6)
*c* (Å)	10.6669 (2)	8.0352 (2)
*α* (^o^)	90	90
*β* (^o^)	90.944 (2)	91.170 (2)
*γ* (^o^)	90	90
*V* (Å^3^)	1235.41 (4)	1420.50 (6)
Z	4	4
Dx (g cm^−3^)	1.321	1.196
Absorption coeff. (mm^−1^)	0.301	0.255
*F* (000)	520	552
Crystal size (mm)	0.12 × 0.07 × 0.03	0.10 × 0.07 × 0.03
Data collection and structure solution		
Data collected	10,651	21,277
Independent reflections	2524	2894
Observed reflections [*I* > *2σ*(*I*)]	2353	2766
*R* (int.)	0.018	0.030
Completeness (%)	99.9	99.9
*T*_max_ */T*_min_	1.000/0.900	1.000/0.779
No. of parameters	147	158
R1[*I* > *2σ*(*I*)]	0.025	0.035
*wR2* (all data)	0.069	0.083
S	1.055	1.093
Largest difference peak and hole (eÅ^−3^)	0.33, − 0.21	0.25, − 0.19

#### Compound 1

**Fig. 7:**
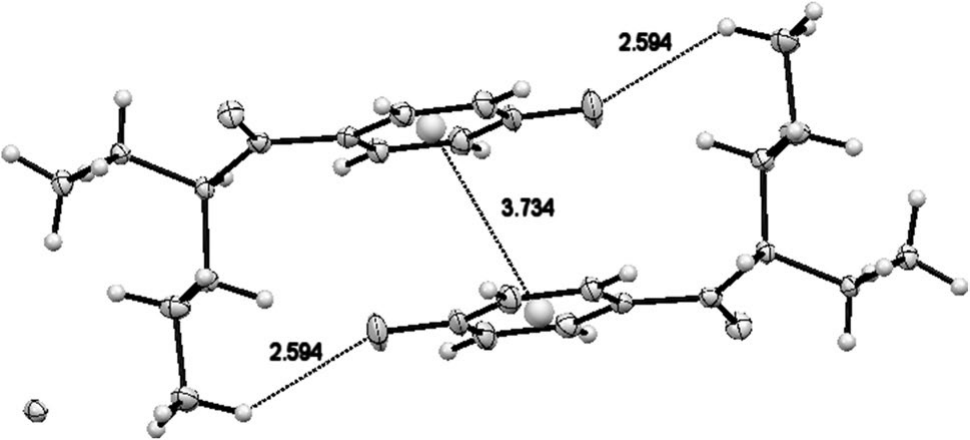
C–H···F and *π*···*π* hydrogen bond motifs in crystal packing of compound **1** (4-FPD hydrochloride)

Two aromatic rings are laid above each other (Fig. 7) with centroid distance equal to 3.734 Å (109.22° angle), in parallel planes separated by 3.473 Å. The shortest distance between oxygen and carbon atoms in the *N*-methyl group of the adjacent molecule was 3.185 Å. The structure featured many other weak hydrogen interactions, for example: NH1A···O1 distance (2.554 Å); NH1B···O1 distance (2.629 Å) and C13H···O1 distance (2.719 Å), all of them shorter than the sum of the van der Waals radii. The shortest distance between the hydrogen atom of the methane group and Cl^−^ ion was 2.606 Å, which is smaller than the sum of the van der Waals radii (2.96 Å). Equally short distances between hydrogen as well as nitrogen atoms and Cl^−^ ions can be found in the structure of this compound (the shortest NH···Cl^−^ = 2.240 Å and N···Cl^−^ = 3.060 Å with the sum of van der Waals radii = 2.95 and 3.30 Å, respectively). Other short distances occurring in the structure of compound **1** are shown in Fig. S9.

#### Compound 2

**Fig. 8:**
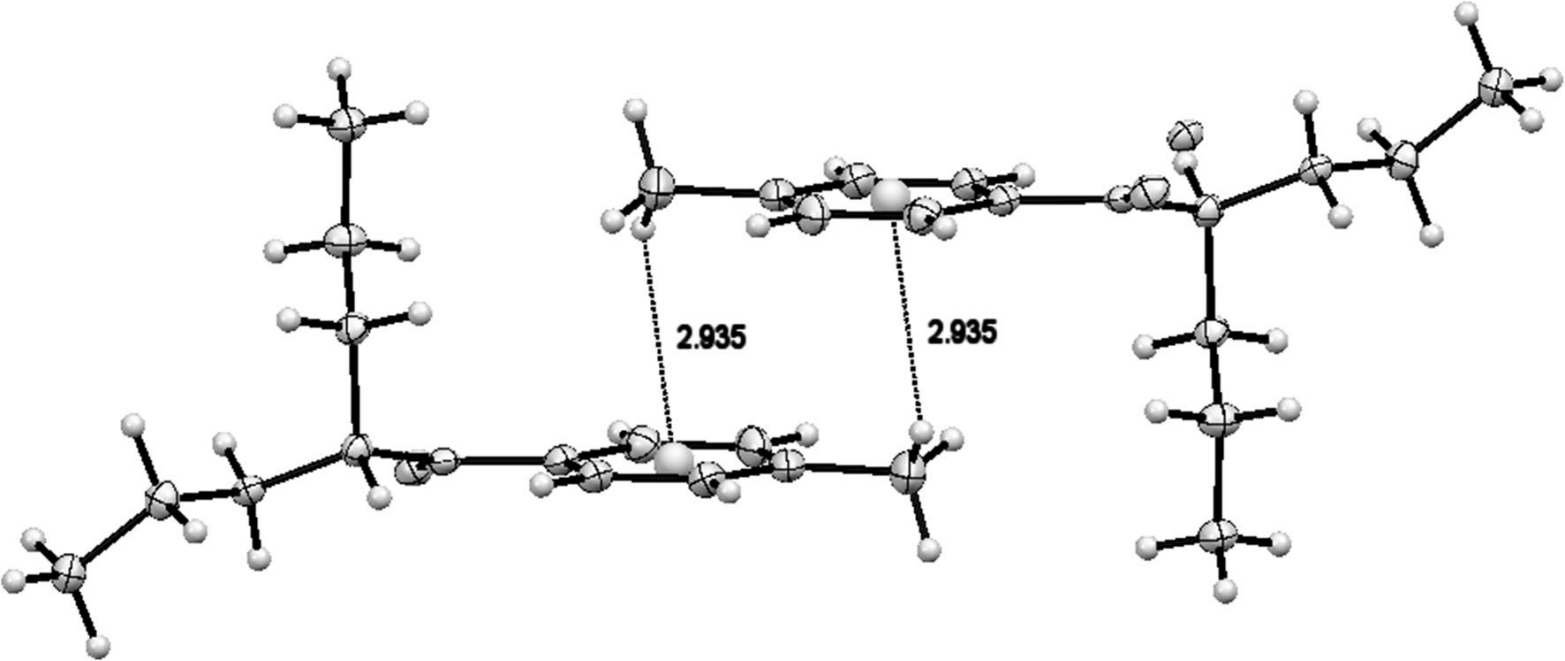
C–H···*π* hydrogen bond motifs in crystal packing of compound **2** (4-MEAP hydrochloride)

The structure of compound **2** lacked strong hydrogen bonds. Weak hydrogen bonds occur inside the molecule in the crystal lattice: O1 ··· H13B distance was 2.825 Å; the distances between oxygen atom and two hydrogen atoms in the amine group were: O1···H1B = 2.574 Å and O1 ··· H1A = 2.576 Å, respectively. Of note, molecules of this compound can form pairs in which the CH···*π*, bond may occur, which is shown in Fig. 8. Lengths of these bonds were: center of aromatic ring—hydrogen in the aromatic methyl group = 2.935 Å (131.91^o^ angle). Aromatic rings lay in parallel planes separated by a distance of 3.460 Å. Several C–H···Cl and N–H···Cl short distances testifying to the presence of interactions between molecules or their fragments are shown in Fig. S10.

## Conclusions

Dynamic growth in the number of psychoactive substances available on the designer drug markets makes it compulsory to obtain analytical data allowing unequivocal identification of these drugs in the fastest possible way. This is of paramount importance for both law enforcement agencies and for forensic toxicologists. In the present paper we report physicochemical characteristics of two cathinone derivatives (4-FPD and 4-MEAP). Both compounds were characterized using an ESI/ion trap MS in MS^2^ and MS^3^ modes, GC–MS, IR, Raman and UV–VIS spectroscopies, DSC, NMR spectroscopy and X-ray crystallography. Our report is linked to the CCDC repository entry of the compound, where its characteristic data can be found, including elementary cell data particularly useful in quick analysis. Although some evidence has been available for 4-MEAP previously (GC–MS, IR and NMR) [19, 20], to our knowledge this study offers the first detailed and comprehensive data for 4-FPD and 4-MEAP.

## Electronic supplementary material

Below is the link to the electronic supplementary material.

Supplementary material 1 (PDF 707 kb)
