# Supplementary material for: Spectroscopic and crystallographic characterization of two cathinone derivatives: 1-(4-fluorophenyl)-2-(methylamino)pentan-1-one (4-FPD) hydrochloride and 1-(4-methylphenyl)-2-(ethylamino)pentan-1-one (4-MEAP) hydrochloride
Source: Forensic Toxicol. 2017 Nov 17;36(1):141–50. doi: 10.1007/s11419-017-0393-6 (PMC5754380; doi:10.1007/s11419-017-0393-6)
Supplement: Supplementary file 1 — Supplementary material 1 (PDF 707 kb) [file 11419_2017_393_MOESM1_ESM.pdf]

Forensic Toxicology – Rojkiewicz *et al.* - Electronic supplementary material

Spectroscopic and crystallographic characterization of two cathinone derivatives: 1-(4-fluorophenyl)-2-(methylamino)pentan-1-one (4-FPD) hydrochloride and 1-(4-methylphenyl)-2-(ethylamino)pentan-1-one (4-MEAP) hydrochloride

Marcin Rojkiewicz[1], Piotr Kuś, Joachim Kusz, Maria Książek
[1] University of Silesia, Institute of Chemistry, Department of Organic Synthesis, 9 Szkolna Street, 40-006 Katowice, Poland

E-mail address: *marcin.rojkiewicz@us.edu.pl* (M. Rojkiewicz)

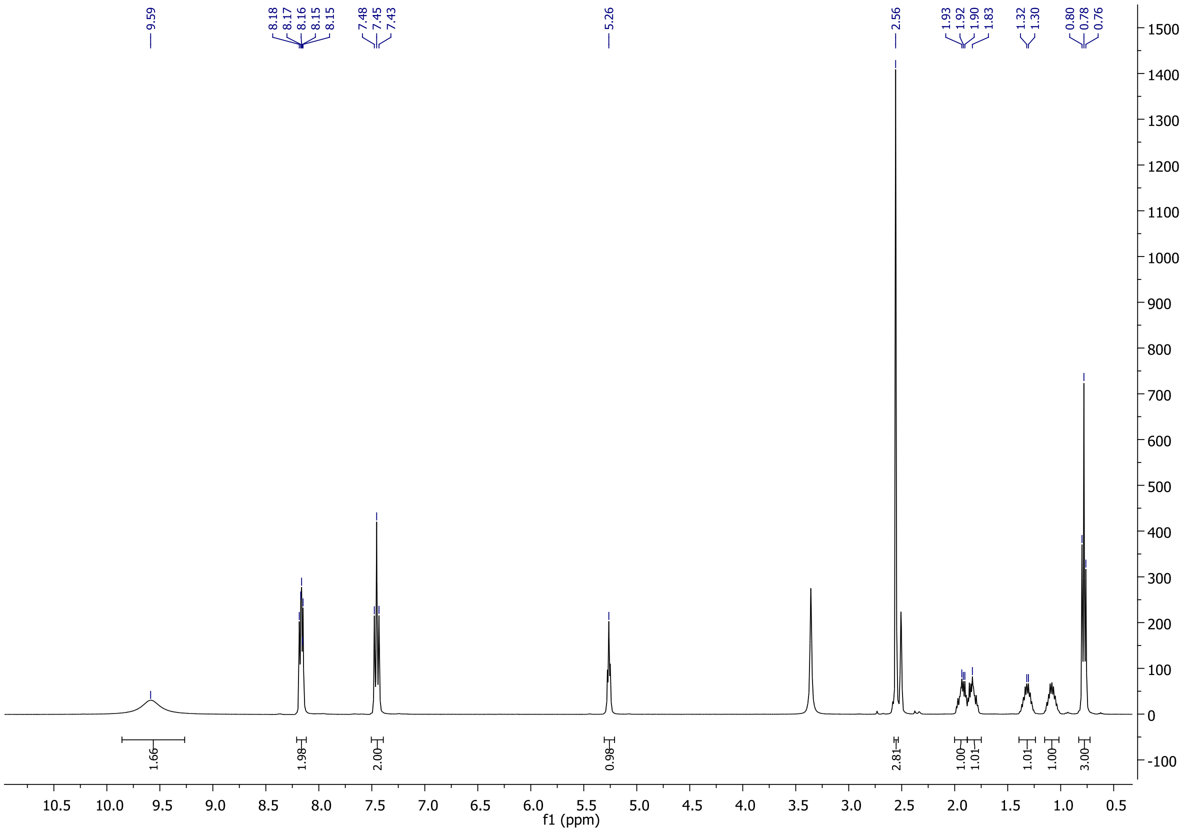

Fig. S1 The ^{1}H nuclear magnetic resonance spectrum for compound **1** (4-FPD)

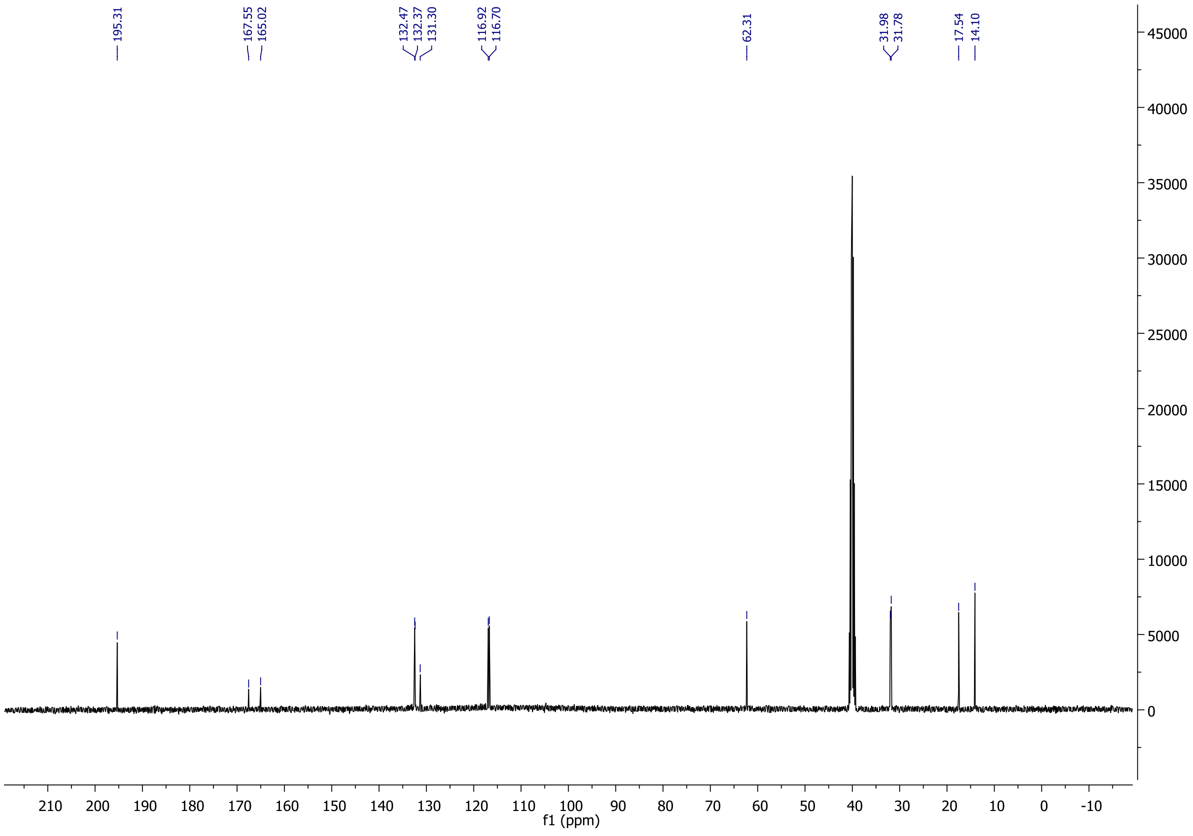

Fig. S2 The ^{13}C nuclear magnetic resonance spectrum for compound **1** (4-FPD)

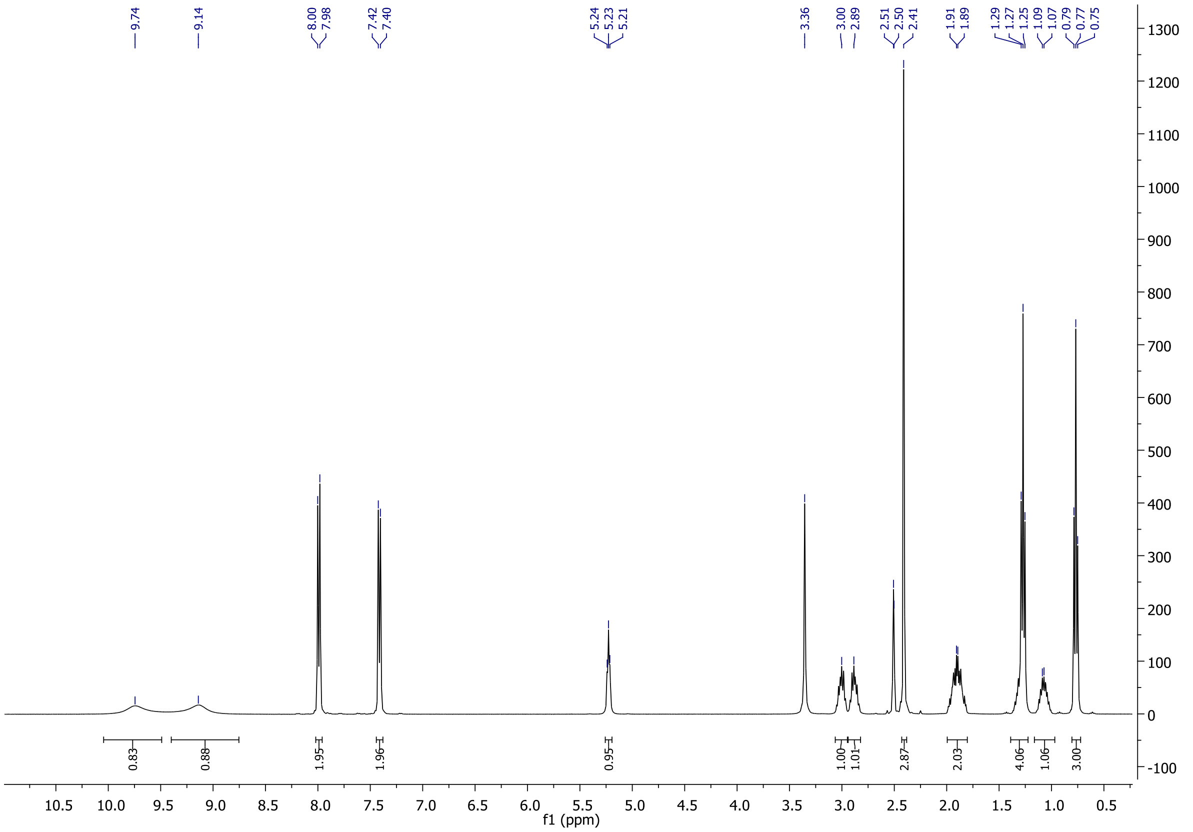

Fig. S3 The ^{1}H nuclear magnetic resonance spectrum for compound **2** (4-MEAP)

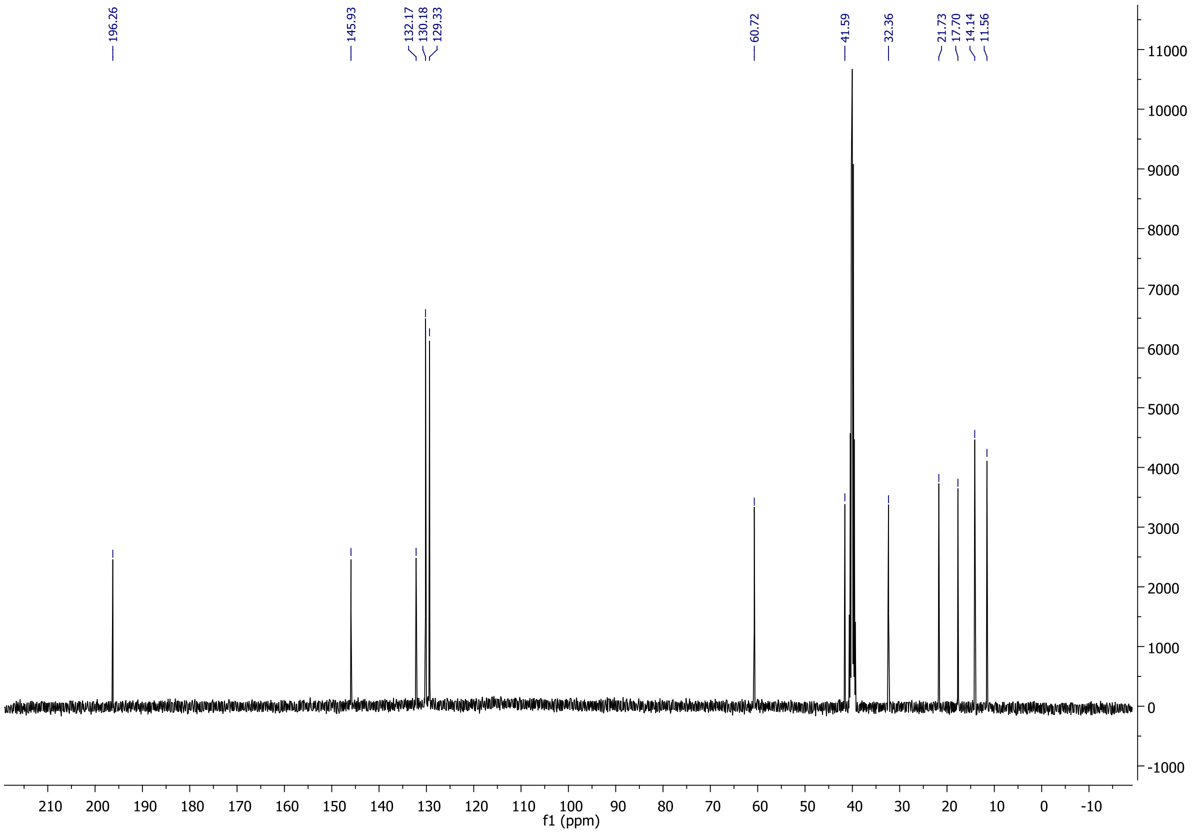

Fig. S4 The ^{13}C nuclear magnetic resonance spectrum for compound **2** (4-MEAP)

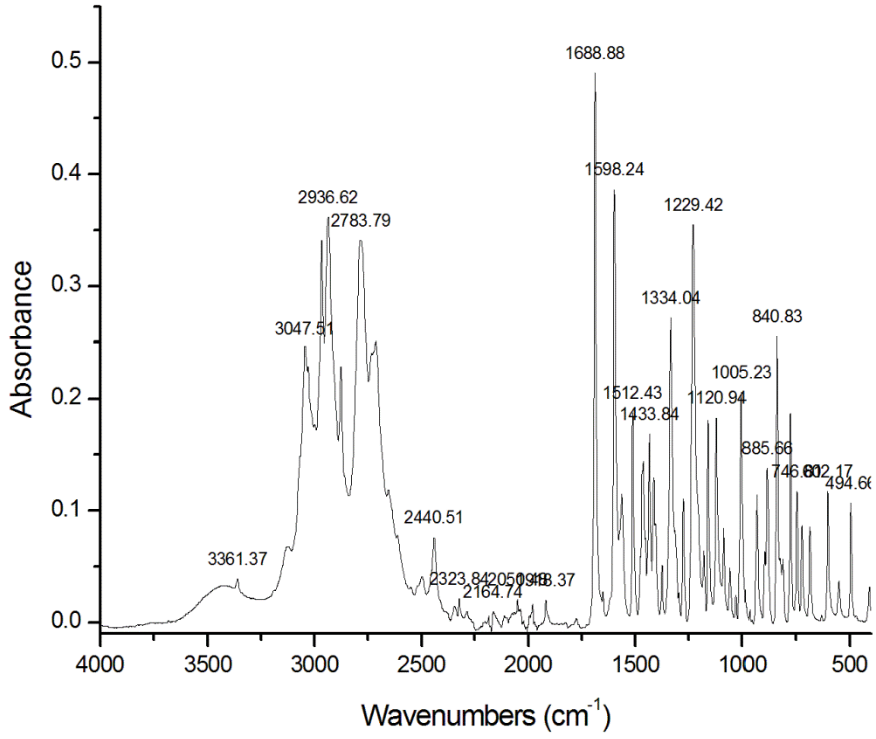

Fig. S5 The IR spectrum for compound **1** (4-FPD)

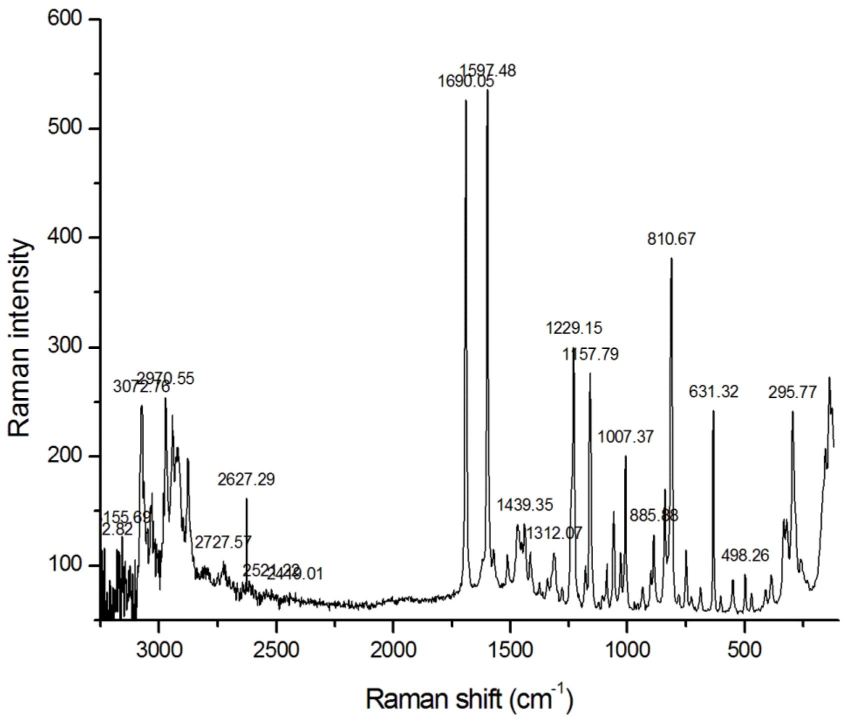

Fig. S6 The Raman spectrum for compound **1** (4-FPD)

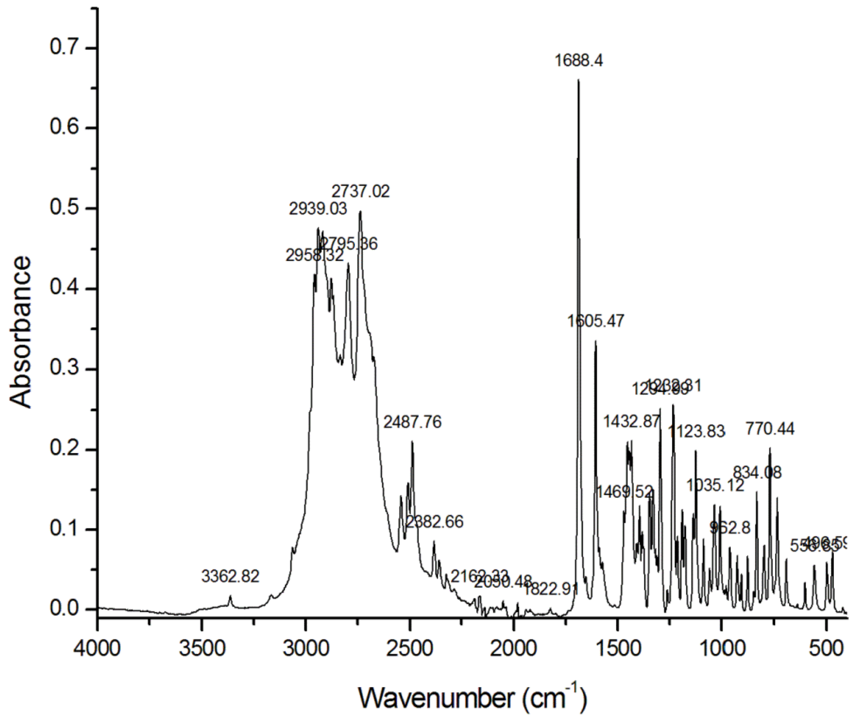

Fig. S7 The IR spectrum for compound **2** (4-MEAP)

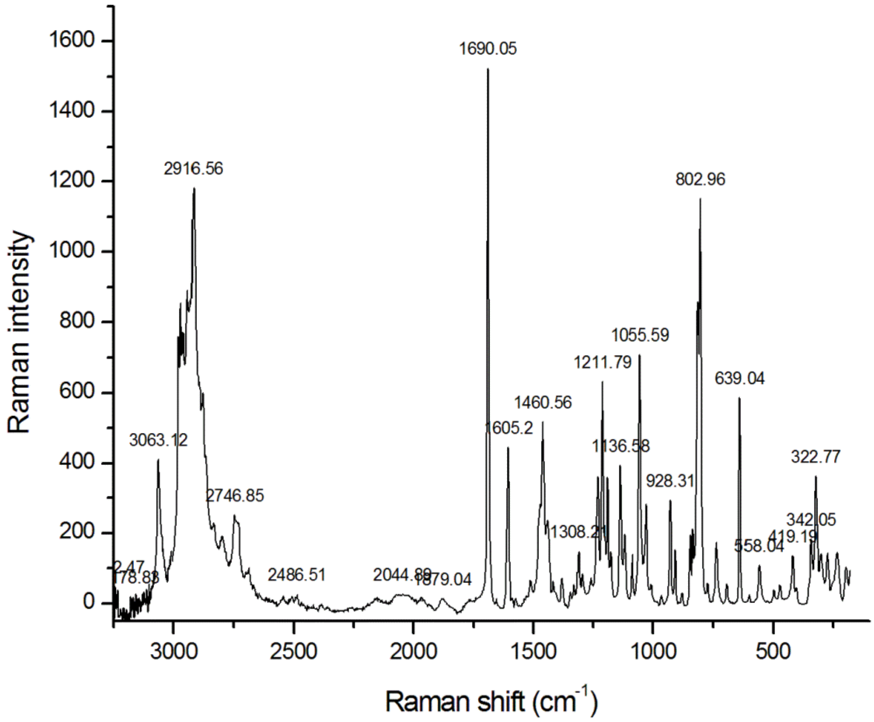

Fig. S8 The Raman spectrum for compound **2** (4-MEAP)

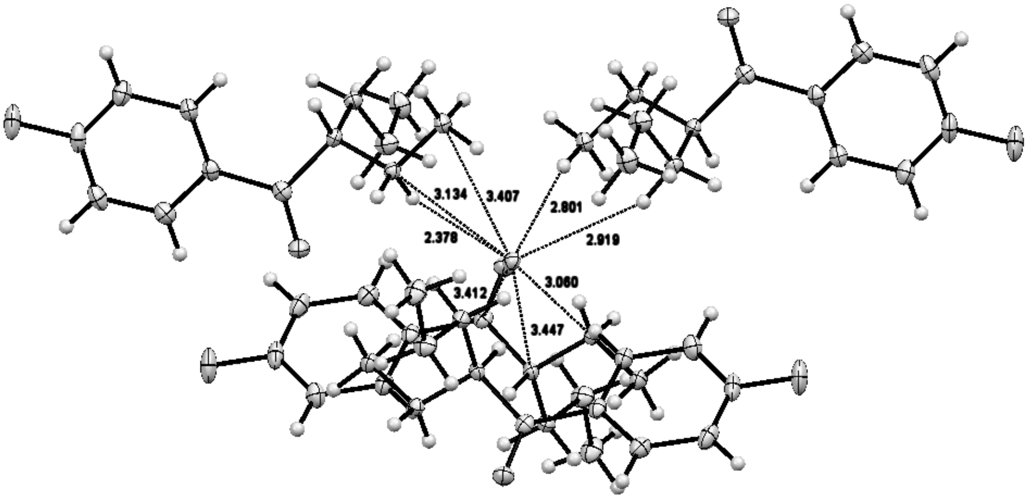

Fig. S9 Short contacts in crystal of compound **1** (4-FPD)

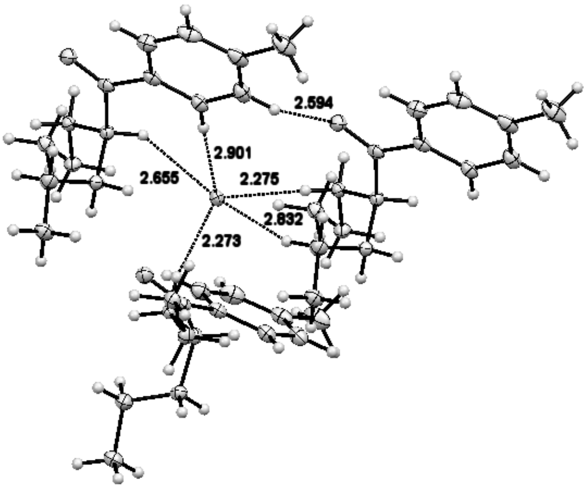

Fig. S10 Short contacts in crystal of compound **2** (4-MEAP)
